# Alzheimer's disease: synaptic dysfunction and Aβ

**DOI:** 10.1186/1750-1326-4-48

**Published:** 2009-11-23

**Authors:** Ganesh M Shankar, Dominic M Walsh

**Affiliations:** 1 Center for Neurologic Diseases, Harvard Medical School and Brigham and Women's Hospital, Boston, MA, USA; 2 Laboratory for Neurodegenerative Research, The Conway Institute for Biomolecular and Biomedical Research, University College Dublin, Belfield, Dublin 4, Republic of Ireland

## Abstract

Synapse loss is an early and invariant feature of Alzheimer's disease (AD) and there is a strong correlation between the extent of synapse loss and the severity of dementia. Accordingly, it has been proposed that synapse loss underlies the memory impairment evident in the early phase of AD and that since plasticity is important for neuronal viability, persistent disruption of plasticity may account for the frank cell loss typical of later phases of the disease. Extensive multi-disciplinary research has implicated the amyloid β-protein (Aβ) in the aetiology of AD and here we review the evidence that non-fibrillar soluble forms of Aβ are mediators of synaptic compromise. We also discuss the possible mechanisms of Aβ synaptotoxicity and potential targets for therapeutic intervention.

## Introduction

Alzheimer's disease (AD) is an irreversible, progressive brain disorder that slowly destroys memory and cognitive skills. It is the most common human dementia and as such confers a huge emotional and economic burden on patients, caregivers and society [1]. Age is the most significant risk factor, with the chance for developing AD doubling every five years after 65 [2,3]. However, the disease can also strike in mid-life and so-called early-onset AD (EOAD) is designated as dementia developing before 65 years old. Fortunately EOAD is rare with an estimated incidence of 4.2 per 100,000 persons in the 45-64 year age group [4]. The cognitive and pathological changes evident in EOAD and late onset AD (LOAD) are highly similar with the former apparently an accelerated form of the more common LOAD. Because many EOAD cases have a strong Mendelian inheritance pattern they have proved instructive in identifying key gene products involved in the disease process. Specifically, autosomal dominant mutations identified in familial AD all appear to converge on altering the processing of the amyloid precursor protein (APP) [5].

The precise onset of clinical AD is difficult to discern but is often manifested as subtle and intermittent deficits in episodic memory. After many months of gradually progressive impairment of first declarative and then also non-declarative memory, other cognitive symptoms appear and slowly advance. Over a further period of years a profound dementia develops that affects multiple cognitive and behavioral spheres [6]. Pathologically, the Alzheimer brain is characterized by atrophy and microscopically there are decreases in the numbers of neuronal cell bodies in the limbic and association cortices and in certain subcortical nuclei [7-9] and numerous amyloid plaques and neurofibrillary tangles litter the cerebrum [10-12]. Many studies have examined the relationship between cognitive impairment and plaque and tangle counts and while, in general, the number of neurofibrillary tangles correlates better with severity of dementia than the number of amyloid plaques, the most robust correlation in the staging of dementia and early AD is the magnitude of synapse loss [13-15].

### Synaptic impairment is likely to be the basis of memory loss in AD

The notion that dementia could result from some sort of synaptic degeneration has been with us for over 100 years and was eloquently expressed by Santiago Ramon y Cajal when he suggested that "dementia could result when synapses between neurons are weakened as a result of a more or less pathological condition, that is, when processes atrophy and no longer form contacts, when cortical mnemonic or association areas suffer partial disorganization" [16]. Quantification using electron microscopy or immunohistochemical staining for synaptic markers has documented significant decreases in synaptic density in the association cortices and hippocampus of AD brain [13,14,17-21] and biochemical and immunohistochemical analysis has revealed a similar loss of both pre-synaptic and post-synaptic components [22]. Moreover, the initial decrease in synapse number and density seems disproportionate to the loss of neuronal cell bodies [13,18,23], suggesting that pruning of synaptic endings may precede frank neuronal loss. Indeed, synaptic degeneration appears to be an early event in pathogenesis with synapse loss evident in patients with early AD and mild cognitive impairment (MCI) [14,15,24].

An important feature of the memory impairment associated with AD and MCI is the selective vulnerability of the ability to consolidate new memories; whereas, the capacity to recall information from the distant past is preserved. This selective impairment of recent memory together with the rapid decay of newly acquired information is reminiscent of the deficits seen in the temporal lobe amnesias and in patients with bilateral hippocampal damage [25,26]. Analogously, extensive experimentation has demonstrated that the memory deficits observed in aged rodents are highly similar to those in animals with bilateral lesioning of the hippocampus and that age-dependent memory impairment is associated with decreased hippocampal synaptic plasticity. Specifically, estimation of the number of axospinous synapses per neuron in the molecular layer of the dentate gyrus revealed that aged rats with impaired spatial memory had fewer simple and perforated synapses than did memory-intact aged or young rats [27-30]. Moreover, old animals with good spatial memory exhibited similar levels of synaptic enhancement as young animals, whereas memory-impaired old rats had impaired enhancement [31]. The prominent pathology evident in the perforant pathway in the early stages of AD [32] and the documented importance of the hippocampus in encoding the memory of recent events [33-35] argue strongly that disconnection of the hippocampal formation is likely to underlie the progressive memory disturbances in AD.

Irrespective of educational or social status few humans will escape the age-dependent memory impairment that is a frequent companion of old age, yet the majority of elderly will not suffer the ignominy of AD. So, what distinguishes MCI and the early periods of AD from non-pathogenic age-dependent memory loss? Clearly the spectrum from healthy ageing to AD is a very broad one, though not necessarily a continuum, with age-dependent deficits often having a strongly frontal involvement [36,37]. However, it has been suggested that AD is an extreme and accelerated version of age-related memory loss [38], but that once this accelerated process is initiated it develops a pathogenic profile not seen in normal ageing. Here we propose that the amyloid β-protein (Aβ) is the molecular accelerant that hastens age-related memory decline and triggers AD pathogenesis.

### Measures of synaptic plasticity provide a useful read-out to assess AD-related pathophysiology

Hebbs postulate describes a basic mechanism for synaptic plasticity wherein an increase in synaptic efficacy arises from the pre-synaptic cell's repeated and persistent stimulation of the post-synaptic cell [39]. Accordingly, long term potentiation (LTP) and long term depression (LTD) are widely used as models of learning and memory and such processes are believed to play important roles in neural circuits of the brain, with effects lasting for hours, days, or longer [34,35,40,41]. Similar to LTP, the induction of hippocampal LTD requires activation of NMDA receptors and/or metabotropic glutamate receptors (mGluR), depending on the experimental conditions [42,43]. Mechanistically, the balance between synapse potentiation versus depression is thought to depend on alterations in cytosolic Ca^2+ ^concentration and the differential activation of certain kinases and phosphatases, such as calcium/calmodulin dependent kinase II (CamKII), calcineurin, and cyclic AMP response element binding protein (CREB) [42,43]. Ultimately, this balance in intraneuronal signalling appears to regulate the stability of the post-synaptic density [44] and the post-synaptic content of AMPA receptors [45,46]. Together, these structural changes dynamically modulate the magnitude of neurotransmission at the synaptic level.

Anatomical studies in normal rodents suggest that the induction of LTP is associated with spine formation and increased spine volume, whereas the induction of LTD results in decreased spine volume and spine elimination [47-49]. Similarly, processing of information for long-term storage requires specific patterns of activity that lead to modification of synapse structure and eventually to change in neural connectivity [50]. Increases in synapse density and stabilization of dendritic spines in the mouse barrel cortex have been described following whisker stimulation, providing insights into structural changes in synapses associated with learning and memory [51]. It is also well-established that synapses throughout the CNS integrate synaptic activity to maintain and regulate receptors and channels at synapses [40]. Without regulation of synapse activity, maintenance of synaptic proteins is lost, leading to excitotoxicity and degradation of stored memory [40]. Consequently, we believe that studies of synaptic plasticity employing both electrophysiological and morphological measures are essential for developing an understanding of how memory mechanisms are disrupted in disease.

### Aβ exists in many different assembly forms

The molecular pathways leading to synapse loss and dysfunction in AD are not well understood, but substantial data indicate that Aβ may be responsible for these affects [52-54]. Over the past 17 years researchers have built up a detailed picture of the natural economy of brain Aβ. The steady state level of Aβ is controlled by its production, degradation and clearance [55,56] and it is proposed that in disease a defect leading to over-production or decreased clearance causes an accumulation of Aβ. This in turn triggers a pathogenic cascade culminating in the cognitive deficits that characterize AD [57]. Like several other disease-associated proteins, Aβ has the ability to self-associate [58], and can form an array of different assembly forms ranging from dimers all the way to aggregates of fibrils [59]. Initially, it was assumed that toxicity was mediated by fibrillar Aβ similar to that present in amyloid plaques. However, the quantity and temporal progression of amyloid plaques do not correlate well with the clinical progression of the disease [20,60,61], thus raising the simple question: if Aβ causes AD, then why doesn't the amount of Aβ in the form of amyloid plaques relate to the severity of dementia?

Recent studies suggest soluble non-fibrillar Aβ assemblies, which go by names such as ADDLs, oligomers, paranuclei, and protofibrils (Table 1) [62-66] may provide the missing link, but as yet the specific form(s) of Aβ which causes injury to neurons *in vivo *has not been identified. Perhaps the most compelling argument in support of a role for non-fibrillar soluble Aβ comes from a common sense inspection of available data. Extensive multi-disciplinary research provides incontrovertible evidence that Aβ plays an important role in AD [57]. This together with the findings that monomer is innocuous and that amyloid plaques alone cannot account for disease has lead many to conclude, if it isn't fibrillar Aβ (akin to that found in amyloid plaques) and it isn't Aβ monomer then it must be some other form of Aβ. Similarly, it seems reasonable that the synaptic and neuronal compromise seen at sites distant from plaques is mediated by an Aβ species that can readily diffuse and access the space in and surrounding the synaptic cleft.

**Table 1:** Water-soluble non-fibrillar Aβ assemblies

Name	Physical Characteristics	Biological activity
Protofibrils*	β-sheet-rich, curvilinear structures of 6-8 nm in diameter and 5-200 nm in length [63,118]. Under certain circumstances canalso form annular structures [160].	Alter neuronal activity [108], block *in vitro *LTP [116] cause memory impairment and phosphorylation of tau [117].

ADDLs	A mixture of monomer and heterogenous high molecular weight oligomers [120] some of which appear similar to small protofibrils [121].	Block *in vitro *LTP [64] and cause loss of synapses and [111] at higher concentrations neuronal death [123].

Globulomers	Formed in the presence of SDS or fatty acids they are β-sheet-rich and by AFM appear as spheres of 1-5 nm [161] and elute from SEC with a molecular weight consistent for a globular protein of ~100 kDa.	Bind to hippocampal neurons, inhibit LTP *in vitro *[162] and block P/Q Ca^2+ ^currents [163].

Spheroids	Spherical structures of 3-20 nm diameter that co-migrate on glycerol gradients with thyroglobulin (669 kDa) [65]. Similar structures have been immunoisolated from human brain [164].	Induce activation of GSK-3β and cell death in cultured neurons [65].

Disulphide cross-linked Aβ	Isolated cysteine linked dimers of Aβ that lack significant secondary structure (O'Nuallain and DMW, unpublished) and behave as true dimers on SEC and analytical ultracentrifugation [77].	Block LTP *in vitro *[77] and *in vivo *[165] and impair the memory of learned behaviour (Cleary and DMW, unpublished).

Cell-derived SDS-stable low-n oligomers	Present in medium from CHO cells expressing human APP and which migrate on SDS-PAGE and SEC with molecular weights consistent for Aβ dimers and trimers [95,105].	Block LTP *in vitro *[104] and *in vivo *[102] and impair the memory of learned behaviour [107].

Aβ56*	An Aβ-immunoreactive species isolated from brains of APP transgenic mice in the presence of 0.01% NP-40 and 0.1% SDS and which migrates on SDS-PAGE and SEC with a molecular weight comparable to a globular protein of 56 kDa [86].	Causes impairment of spatial memory [86].

Brain-derived SDS-stable Aβ dimer	Extracted from human brain using aqueous buffer and remain in solution following high speed centrifugation. These species migrate on SDS-PAGE as dimers, but elute from SEC as true dimers and high molecular weight species [77] (McDonald *et al*. Water- and triton-X100 extracted Aβ is specific for Alzheimer-type dementia, submitted).	Block LTP *in vitro *and *in vivo *and impair the memory of learned behaviour [77].

Besides the forms of Aβ described above a significant number of other Aβ preparations have been studied, but since most of these have not been characterized beyond the use of denaturing electrophoresis or reactivity with certain antibodies they are not dealt with here.

* Protofibrils appear prior to detection of mature amyloid fibrils and thus are considered as pre-fibrillar assemblies.

In recent years biochemical analysis of AD brain have revealed a robust correlation between soluble Aβ levels and the extent of synaptic loss and severity of cognitive impairment [67-70]. But what exactly constitutes soluble Aβ is as yet not well-understood. The term soluble Aβ is an operational definition, embracing all forms of Aβ that remain in aqueous solution following high speed centrifugation of brain extracts [68-71]. Moreover, the origin of soluble Aβ is ambiguous. Extraction of Aβ from brain invariably involves homogenization and consequent cell fracture thus the extracted pool will include truly soluble extracellular Aβ, extracellular Aβ loosely associated or in equilibrium with plaques and a portion of intracellular Aβ. To date, most studies of soluble cerebral Aβ have employed ELISA methods that cannot disclose the aggregation state of the species detected and for the most part appear to preferentially detect Aβ monomer [72-76]. Although the detection methods used provide little information about the assembly state of Aβ the fact that they are not sedimented by ultracentrifugation indicates that they are not mature amyloid fibrils. While a huge amount of data has been gathered concerning the primary sequence of cerebral Aβ only limited attempts have been made to assess the assembly forms of Aβ present in human brain. Using aqueous buffer free of detergents or chaotropes, Kuo and colleagues [71] isolated a range of non-fibrillar forms of Aβ from both AD and control brain. Both sample populations contained a continuous distribution of Aβ species from monomer up to oligomers in excess of 100 kDa, with the major contribution coming from low-n oligomers ranging from dimers to octamers. In agreement with these results we have found that aqueous extracts of AD brain contain a distribution of Aβ species which migrate on non-denaturing size exclusion chromatography (SEC) [77] and glycerol velocity gradient centrifugation (GVGC) over a broad molecular weight range (J. McDonald and DMW unpublished). However, given that Aβ can also bind to other proteins, the molecular weight distribution determined by ultrafiltration, SEC and GVGC cannot be definitively ascribed to homo-oligomers of Aβ.

In a complementary study, McLean and colleagues extracted samples of frontal cortex and putamen in PBS and centrifuged these at 175,000 g for 30 min [68]. Western blot analysis of the supernates from AD brain revealed the presence of variable proportions of monomeric, dimeric and trimeric Aβ species [68]. Such SDS-stable low-n oligomers have also been detected in human CSF by LC-MS [78] and appear to represent highly stable non-covalently associated dimers of Aβ_1-40 _and trimers of either Aβ_6-42 _or Aβ_1-35_. Higher molecular weight SDS-stable assemblies have not been reported in human CSF or soluble extracts of human brain, but whether the dimers and trimers detected represent true low-n oligomers or are breakdown products of larger SDS-unstable assemblies is unclear. Moreover, the presence of SDS-stable dimers and trimers in the soluble fraction of human brain and in extracts of amyloid plaques [68,74,75,79] suggest that in addition to being the earliest mediators of neuronal dysfunction SDS-stable low-n oligomers of Aβ may be the fundamental building blocks of insoluble amyloid deposits.

Transgenic mouse models over-expressing human APP develop many facets of amyloid pathology [80,81] and have been studied in an effort to identify toxic Aβ assemblies [82-87]. Particularly noteworthy evidence supporting soluble forms of Aβ as the principal mediators of neuronal compromise comes from a report using PDAPP mice in which Aβ-mediated deficits of memory were reversed by a single intraperitoneal injection of an anti-Aβ antibody [88]. In these acute (< 24 hr) experiments, brain amyloid burden was not decreased, suggesting that the antibody was acting on soluble, diffusible species of Aβ and that neutralization or clearing these small intermediates allowed rapid improvement in object recognition performance.

Using another well-characterized APP transgenic mouse model, Tg2576, Lesne and colleagues reported that in brain extracts from Tg2576 mice ~42 kDa (nonamer) and ~56 kDa (dodecamer) Aβ species were detected at an age that coincided with the first observed changes in spatial memory [86]. Aβ monomer, trimer and hexamer were seen at earlier time points and hence were not considered to be associated with a deleterious effect on cognition. Indeed, comparison of spatial memory and the levels of Aβ monomer, trimer, hexamer, nonamer and dodecamer revealed that only nonamer and dodecamer levels correlated with impairment of spatial memory. Importantly, ventricular injection of purified dodecamer into normal pre-trained wild-type rats dramatically perturbed the memory of learned behaviour [86,89], thus demonstrating that a soluble, brain-derived form of Aβ can directly mediate brain dysfunction. However, it is unlikely that nonamer and dodecamer alone are the only Aβ assemblies capable of altering brain function. For instance, Kawarabayashi and colleagues reported that the appearance of SDS-stable dimers present in lipid rafts also coincided with impairment of spatial memory [85]. In addition, the same Tg2576 mice demonstrate poor performance in a hippocampus-dependent contextual fear conditioning assay, decreased spine density in the dentate gyrus, and impairment of long term potentiation (LTP) at ages long before the first detection of Aβ dodecamer [86,90,91]. Thus, while the appearance of dodecamers correlate with the impairment of spatial memory in Tg2576 mice, it does not correlate with changes in other forms of memory, nor do dodecamer levels correlate with changes in synaptic form and function. Indeed, very recent data support the presence of multiple bioactive Aβ species in APP transgenic mouse brain [92,93]. In J20 APP transgenic mice, over-expression of neprilysin dramatically reduced total Aβ levels, but did not alter dodecamer and SDS-stable Aβ trimer levels nor did it recover spatial reference learning and memory impairments evident in J20 mice. In addition, changes in hippocampal Fos levels and hyperactivity were attributed to a third unidentified species [93]. Similarly, we found the presence of several different Aβ assemblies in the cerebrum of J20 mice, before, coincident with, and after the onset of detectable synapto-dendritic compromise [94] thus it seems likely that more than one Aβ species mediates the various synaptic deficits evident in APP transgenic mice.

SDS-stable low-n oligomers bearing some similarities to those detected in human and mouse brain have been detected in the conditioned medium (CM) and/or lysates of a variety of cell lines [73,95-100]. Chinese hamster ovarian (CHO) cells that express mutant (V717F) human APP (referred to as 7PA2 cells) produce and secrete low nanomolar amounts of Aβ species that migrate in denaturing SDS-PAGE with molecular weights consistent for Aβ monomer, dimer and trimer [95,101]. Because of the easy maintenance and fast growth rate of 7PA2 cells, 7PA2 CM has become the media of choice to investigate the biological activities of cell-derived SDS-stable low-n Aβ oligomers. 7PA2 medium containing Aβ low-n oligomers has a variety of plasticity and memory impairing effects. It can block LTP *in vivo *[102,103] and *in vitro *[104,105], reduce spine density in cultured neurons [100,106] and decrease the density of synapses *in vivo *(Freir *et al*. Cell-derived Aβ oligomers inhibit synapse remodelling necessary for memory consolidation, submitted). When injected into the lateral ventricle 7PA2 CM or oligomer-containing fractions thereof impair performance on operant lever tasks such as the alternating lever cyclic ratio test [89,107], disrupt working memory in rats tested in a radial-arm maze [99] and show a time-dependent interference with consolidation of avoidance learning (Freir *et al*. Cell-derived Aβ oligomers inhibit synapse remodelling necessary for memory consolidation, submitted).

Importantly, biologically active non-fibrillar, non-monomeric assemblies of synthetic Aβ have also been identified and such species of Aβ have been shown to exert disease relevant toxicities [63,64,94,100,108-112]. Preparations containing toxicity mediating forms of Aβ are frequently referred to as oligomers, however, by and large, the assembly state of these have been poorly defined and it is very difficult to compare Aβ preparations used in different studies. Here we will review some of the most commonly encountered and better characterized Aβ preparations. Protofibrils (PFs) have been characterized using a combination of size exclusion chromatography, quasi-elastic light scattering, electron microscopy (EM) and atomic force microscopy (AFM) and derive their name due to the fact that these structures share some physical similarities with amyloid fibrils but appear prior to the detection of fibrils [62,63]. In solution PFs are highly polydisperse and by EM or AFM range in size from spheres ~5 nm in diameter to curvilinear structures up to 200 nm in length. Their formation is dependent on concentration, pH and ionic strength [113] and they appear to behave as true fibril intermediates in that they can both form fibrils and dissociate to low molecular weight species of Aβ [63,113]. Annular PFs with external and internal diameters of ~8 and ~2 nm have been detected and seem to be particularly well-populated in preparations of Aβ peptide bearing the Arctic mutation (E22G) [114]. Under conditions where there appeared to be little conversion of PFs to fibrils, addition of PFs to rat cortical cultures caused a time-dependent decrease in neuronal viability as measured by LDH release or an increase in Hoechst staining [108,115]. Moreover, PFs caused a dose-dependent inhibition of MTT reduction by primary neuronal cultures that was detectable after only 2 hours of incubation with cells [63], an almost immediate enhancement of electrical activity of neurons [108] and a robust block of LTP [116]. It has also been demonstrated that that certain naturally occurring lipids can cause disassembly of fibrils leading to the formation of protofibrils and that when these so-called "backward PFs" are injected into the ventricle can spread rapidly throughout the brain and alter learned behaviour [117].

Aβ-derived diffusible ligands (ADDLs) which are sometimes simply referred to as Aβ oligomers were first detected and studied by Mary Lambert, Bill Klein and colleagues [64]. Until relatively recently there was little physical definition of ADDLs. The original report simply documented the detection by AFM of imperfect spheres ~5-6 nm in diameter which appeared highly similar to the smallest PF species detected by AFM and EM [62,118,119]. In contrast solution state analysis of ADDLs using SEC, light scattering and analytical ultracentrifugation indicate that the classic "Klein ADDL preparation" contain a mixture of different sized species and include Aβ assemblies which are not well-detected by EM or AFM [120,121]. ADDL preparations contain a huge array of species ranging from monomer to large assemblies with molecular weights approaching a million Daltons [120], however, the relative abundance of the high molecular weight species and monomer appear to vary depending on the precise incubation conditions. For instance, Hepler and colleagues found monomer to be the major species in their ADDL preparation [120], whereas Lauren and colleagues found the high molecular weight species to predominant [121].

ADDLs have been shown to cause neuronal death, block LTP [64,109] and inhibit reduction of MTT by neural cell lines [109,122,123]. When incubated with organotypic mouse brain slice cultures for a 24 h period low concentrations of ADDLs (5 nM) caused ~20% loss in cell number whereas at higher concentrations (500 nM) and brief incubation periods (45-60 minutes) cell loss was not evident but a near complete abrogation of LTP was observed [64,109]. Consistent with their synaptotoxic activity, ADDLs have been shown to avidly bind and decorate dendritic arbors of certain cultured neurons [121,124,125] and to mediate dendritic spine loss [111]. The *in vivo *relevance of ADDLs is suggested by the finding that antibodies (M93 and M94) to synthetic ADDLs prevented Aβ-induced toxicity and that both synthetic Aβ_1-42 _and some forms of brain-derived Aβ bound specifically to the surface of cultured hippocampal neurons [125,126]. These antibodies only weakly reacted with monomer and preferentially recognized assembled forms of Aβ [126], and dot blot analysis of soluble extracts from human AD brain revealed a dramatic increase in M93 immunoreactivity compared to control brain [125]. Similarly, soluble brain extracts from old Tg2576 mice displayed significant anti-ADDL immunoreactivity [127]. However, given the highly heterogeneous nature of the synthetic ADDL immunogen used to generate M93 it is unclear exactly what species of Aβ this antibody recognizes.

Besides the use of ADDLs and PFs a number of studies have employed a variety of other non-fibrillar preparations and found these also to be toxic to cultured neurons [65,128-132]. For example, when Deshpande and colleagues examined the effect of 3 distinct assembly forms of synthetic Aβ, they found that all preparations tested were toxic to primary human cortical neurons, but that the extent and mechanism of toxicity differed [133]. The forms of Aβ investigated were, high molecular weight oligomers (formed as described by [134]) ADDLs and fibrillar Aβ. Low micromolar concentrations (5 μM) of high molecular weight synthetic oligomers caused widespread death within 24 h, whereas similar concentrations of ADDLs took 5-times longer to cause cell loss, and 4-fold higher concentrations of fibrillar Aβ took 10 days to induce only modest cell death. Both high molecular weight oligomers and ADDLs bound rapidly and avidly to synaptic contacts. High molecular weight oligomers caused activation of the mitochondrial death pathway, but activation of this pathway also occurred when *sub-lethal *levels of the same oligomers were used, suggesting that such changes may underlie defective synaptic activity in neurons that are still viable. In contrast, Wogulis and colleagues found that overt neuronal loss required the presence of both unaggregated soluble Aβ and fibrillar Aβ [135]. The apparent conflict between this report and those ascribing toxicity to a specific Aβ species likely results because of the use of relatively long incubation conditions. During prolonged incubation with neurons intermediates have the potential to further associate and transition to higher ordered aggregates and fibrils have the ability to dissociate Thus such experimental formats render it difficult to unambiguously ascribe cytopathological activity to a discrete species. Other studies have reported that the application of sub-lethal concentrations of various non-fibrillar Aβ assemblies can alter neuronal architecture, cause perturbations in axonal transport and reduced cell surface levels of NMDA receptors [130,131,136-139].

The role of low-n oligomers of Aβ in the range of dimer to tetramer is supported by results from studies using peptides bearing disease-associated and design mutations. A mutation associated with EOAD which results in the deletion of glutamic acid 22 produces Aβ which *in vitro *exhibits accelerated oligomerization without fibrillation and which potently blocks LTP [140]. In contrast, design mutations substituting glycine for leucine within the GxxxG repeat motif of Aβ show a greater propensity for aggregation and decrease the abundance of mass spec-detected dimers and trimers relative to wild type Aβ_1-42_. Accordingly, such mutants exhibit reduced toxicity when incubated with cultured neurons [141]. Using a similar approach Harmeier and colleagues found that Aβ_1-42 _peptides containing G33A or G29/33A substitutions formed reduced amounts of low-n oligomers, and that the low-n oligomers formed did not block LTP. This latter finding indicates that aggregation size alone is not the sole determinant of synaptotoxicity, but that the solution structure of assemblies is also critical. Interestingly, in comparison to wild type Aβ, over-expression of these peptides in drosophila resulted in lower cell loss and reduced eye degeneration [142].

In summary, studies using synthetic Aβ peptides, Aβ-containing cell culture medium, APP transgenic mouse and human brain demonstrate that Aβ toxicity is a complex and multifaceted phenomenon that may be induced by multiple assembly forms of Aβ and which can result in a variety of effects ranging from reversible changes in synaptic form and function all the way to frank neuronal loss.

### Mechanisms of Aβ-mediated synaptic dysfunction

How Aβ mediates its effects on synaptic plasticity may take many years to fully understand, but already we know that it is likely to involve three different levels. The first and most important mechanism impugns a toxic gain of function for Aβ which results due to self-association and attainment of new structures capable of novel interactions that lead to impaired plasticity. The other two scenarios predicate that Aβ has a normal physiological role. On the one hand insufficient Aβ could lead to a loss of normal function, whereas excess Aβ may precipitate dysfunction.

A growing body of literature supports a physiological role for Aβ in normal synapse function. For instance, in organotypic hippocampal slices, β-secretase activity is increased by synaptic activity and the resulting Aβ peptides depress excitatory transmission through AMPA and NMDA receptors, suggesting a role for Aβ in homeostatic plasticity [143]. Indeed in APP transgenic mouse brain there is a strong positive correlation between synaptic activity and the concentration of Aβ in the interstitial fluid [56] and in humans cerebral Aβ concentration increases as neuronal function and mental status recover in patients with traumatic brain injury [144]. Aβ may also have a role in regulating well-described forms of synaptic plasticity. Specifically, exogenous application of Aβ partially occludes mGluR-dependent LTD, suggesting shared pathways that elicit and modulate, this form of endogenous synaptic plasticity [145]. It has also been suggested that Aβ is important for neuronal survival [146] and it has been shown that picomolar concentrations of human Aβ_1-42 _increase the magnitude of LTP generated in hippocampus by recruiting an α7-acetylcholine receptors pathway [147]. While a physiologic role for Aβ can be surmised from such studies, the Aβ assembly form responsible for these effects is not known. However, it seems likely that monomeric Aβ would mediate these effects, not least since monomer would be the predominant form of Aβ present in freshly reconstituted synthetic peptide or cortical Aβ. Consequently, pathologic effects on synapse physiology may not only arise from the appearance of higher order Aβ assemblies, which assume a toxic gain of function, but also rising level of monomeric Aβ. Thus increased concentrations of Aβ could lead to synaptic dysregulation mediate by abnormally high levels of monomer and the formation of toxic oligomers. For instance it seems plausible that the increase in non-convulsive seizures observed in APP transgenic mice result due to an Aβ-dependent imbalance of excitatory and inhibitory activity [148]. In this scenario Aβ promotes neuronal over-excitability, which results in GABAergic sprouting of inhibitory synapses as a compensatory mechanism. Similarly, using multiphoton imaging of intraneuronal calcium fluctuations high focal levels of Aβ were shown to increase heterogeneity in the excitability of neurons within 60 μm of amyloid plaques [149]. Moreover, dendritic spine loss observed within 20 μm of amyloid plaques in the Tg2576 APP transgenic mouse provides a structural correlate to the physiologic findings [150].

Interactions between Aβ and various receptors have been shown through biochemical and pharmacologic techniques. Given the profound loss of cholinergic transmission in AD nicotinic and muscarinic acetylcholine receptors have drawn considerable attention. Synthetic Aβ has been shown to bind the calcium permeable α7 nicotinic acetylcholine receptors with high affinity [151]. Functionally, this interaction has been proposed to account for the internalization of NMDA receptors through a calcineurin dependent pathway [137,152]. Because these studies focus on post-synaptic cholinergic transmission, it is unclear whether interactions with acetylcholine receptor signalling directly account for the disruption of pre-synaptic cholinergic projections in AD, such as those extending from the nucleus basalis of Meynert.

Hebbian mechanisms of synapse modulation often implicate involvement of NMDA receptors, which transduce the level of synaptic activity into a calcium signal that can initiate an array of signalling pathways. The current-voltage relationship that is generated by the magnesium block of this receptor is a key determinant in the amount of calcium that enters upon glutamate binding. Whether NMDA receptor activation will result in LTP or LTD appears to be dependent on the amplitude and kinetics of the calcium transient through the channel [153,154]. A number of studies have reported that the effects of Aβ on the viability, morphology and physiology of neurons are dependent on NMDA receptor activation [106,111,155]. Memantine, an activity dependent NMDA receptor antagonist is used for the treatment of AD. Initially it was proposed to mitigate glutamate excitotoxicity, but it may also block more subtle effects of NMDA receptor activation that lead to synaptic depression and loss.

Activation of metabotropic glutamate receptors (mGluR) recruits a number of signalling pathways (such as p38 MAP kinase or ERK), stimulates release of intracellular calcium stores through generation of inositol triphosphate, or modulates associated ionic channels. These effects may result in post-synaptic AMPA receptor endocytosis [156] and decreased pre-synaptic neurotransmitter release probability [157], both of which decrease synaptic strength. Understanding the contribution of these receptors to Aβ-mediated synaptic depression is difficult because of the high variability in mechanisms linked to mGluRs across brain regions and developmental time periods. However, various groups have reported that Aβ mediates synaptic depression and loss through activation of group I mGluRs with p38 MAP kinase and calcineurin as downstream effectors [77,94,104,145,152].

We have found that Aβ oligomers from a variety of sources can facilitate LTD through both mGluRs and NMDA receptors and that this appears to involve dysregulation of the neuronal glutamate transporter [94]. A role for aberrant glutamate transport is supported by *in vivo *microdialysis experiments in which micro-injection of oligomer-containing 7PA2 CM caused a rapid and massive increase in hippocampal interstitial fluid glutamate [158].

In addition to receptors well-known for their involvement in synaptic plasticity, Aβ also appears to engage with other synapse proteins. For example, ADDLs appear to perturb insulin signalling by inducing preferential somatic distribution of the receptor via an NMDAR-dependent pathway [159]. Similarly, non-infectious conformations of the cellular prion protein (PrP) have also been demonstrated to bind ADDLs and the role of PrP as a disease-relevant receptor for Aβ is supported by the finding that knock-out of PrP rescues the block of LTP mediated by ADDLs [121].

But how can the myriad interactions demonstrated between Aβ and several classes of receptors be explained? One possibility is that these reports are all correct, but that Aβ does not directly target a single receptor *per se*, but rather it interacts with synaptic membranes and influences the expression and distribution of receptors thus mimicking the same effect as if Aβ had directly bound and antagonized the effected receptors. Another possibility lies in the diversity of the different assembly forms and conformations of Aβ used. It is quite possible that different Aβ assemblies or conformation have different targets thus depending on the form of Aβ used one group can report activation of the insulin receptor and another antagonism of α7 nicotinic acetylcholine receptors.

### Therapeutic targeting of synaptotoxic Aβ oligomers

Although our understanding of how and what forms of Aβ mediate synaptic dysfunction is incomplete, there is a growing consensus that soluble non-fibrillar Aβ interacts either directly or indirectly with one or more receptors initiating transduction mechanisms that result in decreased plasticity (see appendix 1). If correct, this information already provides for the rational design of disease-modifying therapeutics. Plausible approaches include neutralizing one or more forms of synaptotoxic, non-fibrillar Aβ, and preventing Aβ-mediated perturbations of receptors. As our understanding of the mechanisms contributing to synapse dysfunction continues to develop so too additional therapeutic targets are likely be revealed. In this regard the application of unbiased paradigms in which synaptic alterations are delineated following exposure to well defined soluble Aβ assembly forms should yield novel insights on the pathogenesis of synapse loss (see appendix 2). This information may not only assist in drug design to halt neurodegeneration in AD, but may also promote synapse regeneration. Thus the dream of curing AD may be realized not by just preventing neuronal death, but by actively promoting synaptic connectivity.

## Competing interests

DMW is a shareholder and member of the scientific advisory board of Senexis, plc.

## Authors' contributions

DMW designed the layout and content of the review and DMW and GMS co-wrote the text.

## Appendix 1

### Key observations

1. Synaptic loss is an early and invariant feature of AD the extent of which correlates closely with severity of dementia.

2. MCI and early stage AD have a strong amnestic presentation implicating dysfunction of hippocampal and medial temporal lobe circuitry.

3. Soluble forms of Aβ present in human brain correlate well with synaptic loss and dementia status.

4. Synaptic plasticity as measured by changes in LTP and spine density are perturbed in transgenic animals models of AD and are associated with impaired spatial learning and memory.

5. Changes in plasticity and memory often occur at intervals before transgenic animals have detectable amyloid deposits and can be reversed by acute treatment with anti-Aβ antibodies.

6. Synthetic, cell culture- and brain-derived Aβ inhibits LTP, facilitates LTD, reduces spine and synaptic densities and impairs the memory of learned behaviour.

## Appendix 2

### Critical next steps

1. Employ sophisticated biophysical techniques to determine the size and structure of synaptotoxic human brain-derived Aβ.

2. Identify the cellular and molecular targets of Aβ oligomers.

3. Elucidate the mechanism by which Aβ mediates synaptic compromise: delineating whether these effects involve disruption of receptors secondary to membrane destabilization or are a result of direct Aβ receptor binding.

4. Determine if and how Aβ oligomers alter tau aggregation and/or phosphorylation and how this is linked to neuronal loss.

## References

[B1] Ferri CP Prince M Brayne C Brodaty H Fratiglioni L Ganguli M Hall K Hasegawa K Hendrie H Huang Y Jorm A Mathers C Menezes PR Rimmer E Scazufca M Global prevalence of dementia: a Delphi consensus study Lancet 2005 366 2112 2117 10.1016/S0140-6736(05)67889-0 PMC2850264 16360788

[B2] Kawas CH Clinical practice. Early Alzheimer'sdisease N Engl J Med 2003 349 1056 1063 10.1056/NEJMcp022295 12968090

[B3] Nussbaum RL Ellis CE Alzheimer's disease and Parkinson's disease N Engl J Med 2003 348 1356 1364 10.1056/NEJM2003ra020003 12672864

[B4] Mercy L Hodges JR Dawson K Barker RA Brayne C Incidence of early-onset dementias in Cambridgeshire, United Kingdom Neurology 2008 71 1496 1499 10.1212/01.wnl.0000334277.16896.fa 18981371

[B5] Selkoe DJ Alzheimer's disease: genes, proteins, and therapy Physiol Rev 2001 81 741 766 10.1152/physrev.2001.81.2.741 11274343

[B6] Romanelli MF Morris JC Ashkin K Coben LA Advanced Alzheimer's disease is a risk factor for late-onset seizures Arch Neurol 1990 47 847 850 10.1001/archneur.1990.00530080029006 2375689

[B7] Khachaturian ZS Diagnosis of Alzheimer's disease Arch Neurol 1985 42 1097 1105 10.1001/archneur.1985.04060100083029 2864910

[B8] Gomez-Isla T Hollister R West H Mui S Growdon JH Petersen RC Parisi JE Hyman BT Neuronal loss correlates with but exceeds neurofibrillary tangles in Alzheimer's disease Ann Neurol 1997 41 17 24 10.1002/ana.410410106 9005861

[B9] Uylings HB de Brabander JM Neuronal changes in normal human aging and Alzheimer's disease Brain Cogn 2002 49 268 276 10.1006/brcg.2001.1500 12139954

[B10] Alzheimer A Über einen eigenartigen schweren Erkrankungsprozeβ der Hirnrinde Neurologisches Centralblatt 1906 23 1129 1136

[B11] Terry RD The fine structure of neurofibrillary tangles in Alzheimer's disease J Neuropathol Exp Neurol 1963 22 629 642 10.1097/00005072-196310000-00005 14069842

[B12] Kidd M Alzheimer's disease - An electron microscopical study Brain 1964 87 307 320 10.1093/brain/87.2.307 14188276

[B13] Davies CA Mann DM Sumpter PQ Yates PO A quantitative morphometric analysis of the neuronal and synaptic content of the frontal and temporal cortex in patients with Alzheimer's disease J Neurol Sci 1987 78 151 164 10.1016/0022-510x(87)90057-8 3572454

[B14] Masliah E Mallory M Alford M DeTeresa R Hansen LA McKeel DW Jr Morris JC Altered expression of synaptic proteins occurs early during progression of Alzheimer's disease Neurology 2001 56 127 129 10.1212/wnl.56.1.127 11148253

[B15] Scheff SW Price DA Schmitt FA DeKosky ST Mufson EJ Synaptic alterations in CA1 in mild Alzheimer disease and mild cognitive impairment Neurology 2007 68 1501 1508 10.1212/01.wnl.0000260698.46517.8f 17470753

[B16] Cajal Ry Degeneration and regeneration of the nervous system 1928 Oxford Press, London

[B17] Bertoni-Freddari C Fattoretti P Meier-Ruge W Ulrich J Computer-assisted morphometry of synaptic plasticity during aging and dementia Pathol Res Pract 1989 185 799 802 10.1016/S0344-0338(89)80243-2 2626392

[B18] DeKosky ST Scheff SW Synapse loss in frontal cortex biopsies in Alzheimer's disease: correlation with cognitive severity Ann Neurol 1990 27 457 464 10.1002/ana.410270502 2360787

[B19] Masliah E Cole G Shimohama S Hansen L DeTeresa R Terry RD Saitoh T Differential involvement of protein kinase C isozymes in Alzheimer's disease J Neurosci 1990 10 2113 2124 10.1523/JNEUROSCI.10-07-02113.1990 PMC6570390 2376771

[B20] Terry RD Masliah E Salmon DP Butters N DeTeresa R Hill R Hansen LA Katzman R Physical basis of cognitive alterations in Alzheimer's disease: synapse loss is the major correlate of cognitive impairment Ann Neurol 1991 30 572 580 10.1002/ana.410300410 1789684

[B21] Sze CI Troncoso JC Kawas C Mouton P Price DL Martin LJ Loss of the presynaptic vesicle protein synaptophysin in hippocampus correlates with cognitive decline in Alzheimer disease J Neuropathol Exp Neurol 1997 56 933 944 10.1097/00005072-199708000-00011 9258263

[B22] Reddy PH Mani G Park BS Jacques J Murdoch G Whetsell W Jr Kaye J Manczak M Differential loss of synaptic proteins in Alzheimer's disease: implications for synaptic dysfunction J Alzheimers Dis 2005 7 103 117 discussion 173-180. 10.3233/jad-2005-7203 15851848

[B23] Bertoni-Freddari C Fattoretti P Casoli T Caselli U Meier-Ruge W Deterioration threshold of synaptic morphology in aging and senile dementia of Alzheimer's type Anal Quant Cytol Histol 1996 18 209 213 8790834

[B24] Scheff SW Price DA Schmitt FA Mufson EJ Hippocampal synaptic loss in early Alzheimer's disease and mild cognitive impairment Neurobiol Aging 2006 27 1372 1384 10.1016/j.neurobiolaging.2005.09.012 16289476

[B25] Scoville WB Milner B Loss of recent memory after bilateral hippocampal lesions J Neurol Neurosurg Psychiatry 1957 20 11 21 10.1136/jnnp.20.1.11 PMC497229 13406589

[B26] Zola-Morgan S Squire LR Amaral DG Human amnesia and the medial temporal region: enduring memory impairment following a bilateral lesion limited to field CA1 of the hippocampus J Neurosci 1986 6 2950 2967 10.1523/JNEUROSCI.06-10-02950.1986 PMC6568782 3760943

[B27] deToledo-Morrell L Geinisman Y Morrell F Age-dependent alterations in hippocampal synaptic plasticity: relation to memory disorders Neurobiol Aging 1988 9 581 590 10.1016/s0197-4580(88)80117-9 3062469

[B28] Geinisman Y de Toledo-Morrell L Morrell F Loss of perforated synapses in the dentate gyrus: morphological substrate of memory deficit in aged rats Proc Natl Acad Sci USA 1986 83 3027 3031 10.1073/pnas.83.9.3027 PMC323440 3458260

[B29] Geinisman Y de Toledo-Morrell L Morrell F Aged rats need a preserved complement of perforated axospinous synapses per hippocampal neuron to maintain good spatial memory Brain Res 1986 398 266 275 10.1016/0006-8993(86)91486-1 3801904

[B30] Geinisman Y deToledo-Morrell L Morrell F Persina IS Rossi M Age-related loss of axospinous synapses formed by two afferent systems in the rat dentate gyrus as revealed by the unbiased stereological dissector technique Hippocampus 1992 2 437 444 10.1002/hipo.450020411 1308200

[B31] Barnes CA Memory deficits associated with senescence: a neurophysiological and behavioral study in the rat J Comp Physiol Psychol 1979 93 74 104 10.1037/h0077579 221551

[B32] Hyman BT Van Hoesen GW Kromer LJ Damasio AR Perforant pathway changes and the memory impairment of Alzheimer's disease Ann Neurol 1986 472 481 10.1002/ana.410200406 3789663

[B33] Geinisman Y Age-related decline in memory function: is it associated with a loss of synapses? Neurobiol Aging 1999 20 353 356 discussion 359-360. 10.1016/s0197-4580(99)00072-x 10588585

[B34] Morris RG Long-term potentiation and memory Philos Trans R Soc Lond B Biol Sci 2003 358 643 647 10.1098/rstb.2002.1230 PMC1693171 12740109

[B35] Lynch MA Long-term potentiation and memory Physiol Rev 2004 84 87 136 10.1152/physrev.00014.2003 14715912

[B36] Buckner RL Memory and executive function in aging and AD: multiple factors that cause decline and reserve factors that compensate Neuron 2004 44 195 208 10.1016/j.neuron.2004.09.006 15450170

[B37] Persson J Nyberg L Lind J Larsson A Nilsson LG Ingvar M Buckner RL Structure-function correlates of cognitive decline in aging Cereb Cortex 2006 16 907 915 10.1093/cercor/bhj036 16162855

[B38] Mesulam MM Neuroplasticity failure in Alzheimer's disease: bridging the gap between plaques and tangles Neuron 1999 24 521 529 10.1016/s0896-6273(00)81109-5 10595506

[B39] Hebb DO A Neuropsychological Theory 1949 Lawrence Erlbaum Associates, Mahwah N, J

[B40] Malenka RC Bear MF LTP and LTD: an embarrassment of riches Neuron 2004 44 5 21 10.1016/j.neuron.2004.09.012 15450156

[B41] Whitlock JR Heynen AJ Shuler MG Bear MF Learning induces long-term potentiation in the hippocampus Science 2006 313 1093 1097 10.1126/science.1128134 16931756

[B42] Kemp N Bashir ZI Long-term depression: a cascade of induction and expression mechanisms Prog Neurobiol 2001 65 339 365 10.1016/s0301-0082(01)00013-2 11527572

[B43] Citri A Malenka RC Synaptic plasticity: multiple forms, functions, and mechanisms Neuropsychopharmacology 2008 33 18 41 10.1038/sj.npp.1301559 17728696

[B44] Colbran RJ Targeting of calcium/calmodulin-dependent protein kinase II Biochem J 2004 378 1 16 10.1042/BJ20031547 PMC1223945 14653781

[B45] Kessels HW Kopec CD Klein ME Malinow R Roles of stargazin and phosphorylation in the control of AMPA receptor subcellular distribution Nat Neurosci 2009 12 888 896 10.1038/nn.2340 PMC3922706 19543281

[B46] Kessels HW Malinow R Synaptic AMPA receptor plasticity and behavior Neuron 2009 61 340 350 10.1016/j.neuron.2009.01.015 PMC3917551 19217372

[B47] Matsuzaki M Honkura N Ellis-Davies GC Kasai H Structural basis of long-term potentiation in single dendritic spines Nature 2004 429 761 766 10.1038/nature02617 PMC4158816 15190253

[B48] Zhou Q Homma KJ Poo MM Shrinkage of dendritic spines associated with long-term depression of hippocampal synapses Neuron 2004 44 749 757 10.1016/j.neuron.2004.11.011 15572107

[B49] Bastrikova N Gardner GA Reece JM Jeromin A Dudek SM Synapse elimination accompanies functional plasticity in hippocampal neurons Proc Natl Acad Sci USA 2008 105 3123 3127 10.1073/pnas.0800027105 PMC2268595 18287055

[B50] Lamprecht R LeDoux J Structural plasticityand memory Nat Rev Neurosci 2004 5 45 54 10.1038/nrn1301 14708003

[B51] Holtmaat A Wilbrecht L Knott GW Welker E Svoboda K Experience-dependent and cell-type-specific spine growth in the neocortex Nature 2006 441 979 983 10.1038/nature04783 16791195

[B52] Klein WL Krafft GA Finch CE Targeting small Abeta oligomers: the solution to an Alzheimer's disease conundrum? Trends Neurosci 2001 24 219 224 10.1016/s0166-2236(00)01749-5 11250006

[B53] Selkoe DJ Alzheimer's disease is a synaptic failure Science 2002 298 789 791 10.1126/science.1074069 12399581

[B54] Walsh DM Selkoe DJ Deciphering the molecular basis of memory failure in Alzheimer's disease Neuron 2004 44 181 193 10.1016/j.neuron.2004.09.010 15450169

[B55] Selkoe DJ Clearing the brain's amyloid cobwebs Neuron 2001 32 177 180 10.1016/s0896-6273(01)00475-5 11683988

[B56] Cirrito JR Yamada KA Finn MB Sloviter RS Bales KR May PC Schoepp DD Paul SM Mennerick S Holtzman DM Synaptic activity regulates interstitial fluid amyloid-beta levels in vivo Neuron 2005 48 913 922 10.1016/j.neuron.2005.10.028 16364896

[B57] Hardy J Selkoe DJ The amyloid hypothesis of Alzheimer's disease: progress and problems on the road to therapeutics Science 2002 297 353 356 10.1126/science.1072994 12130773

[B58] Powers ET Morimoto RI Dillin A Kelly JW Balch WE Biological and chemical approaches to diseases of proteostasis deficiency Annu Rev Biochem 2009 78 959 991 10.1146/annurev.biochem.052308.114844 19298183

[B59] Walsh DM Selkoe DJ A beta oligomers - a decade of discovery J Neurochem 2007 101 1172 1184 10.1111/j.1471-4159.2006.04426.x 17286590

[B60] Katzman R Alzheimer's disease N Engl J Med 1986 314 964 973 10.1056/NEJM198604103141506 2870433

[B61] Dickson DW Crystal HA Bevona C Honer W Vincent I Davies P Correlations of synaptic and pathological markers with cognition of the elderly Neurobiol Aging 1995 16 285 298 discussion 298-304. 10.1016/0197-4580(95)00013-5 7566338

[B62] Harper JD Wong SS Lieber CM Lansbury PT Jr Observation of metastable Aβ amyloid protofibrils by atomic force microscopy Chem & Biol 1997 4 119 125 10.1016/s1074-5521(97)90255-6 9190286

[B63] Walsh DM Hartley DM Kusumoto Y Fezoui Y Condron MM Lomakin A Benedek GB Selkoe DJ Teplow DB Amyloid beta-protein fibrillogenesis. Structure and biological activity of protofibrillar intermediates J Biol Chem 1999 274 25945 25952 10.1074/jbc.274.36.25945 10464339

[B64] Lambert MP Barlow AK Chromy BA Edwards C Freed R iosatos M Morgan TE Rozovsky I Trommer B Viola KL Wals P Zhang C Finch CE Krafft GA Klein WL Diffusible, nonfribrillar ligands derived from Aβ_1-42 _are potent central nervous system neurotoxins Proc Natl Acad Sci USA 1998 95 6448 6453 10.1073/pnas.95.11.6448 PMC27787 9600986

[B65] Hoshi M Sato M Matsumoto S Noguchi A Yasutake K Yoshida N Sato K Spherical aggregates of beta-amyloid (amylospheroid) show high neurotoxicity and activate tau protein kinase I/glycogen synthase kinase-3beta Proc Natl Acad Sci USA 2003 100 6370 6375 10.1073/pnas.1237107100 PMC164453 12750461

[B66] Bitan G Kirkitadze MD Lomakin A Vollers SS Benedek GB Teplow DB Amyloid β-protein (Aβ) assembly: Aβ 40 and Aβ 42 oligomerize through distinct pathways Proc Natl Acad Sci USA 2003 100 330 335 10.1073/pnas.222681699 PMC140968 12506200

[B67] Lemere CA Spooner ET Leverone JF Mori C Clements JD Intranasal immunotherapy for the treatment of Alzheimer's disease: Escherichia coli LT and LT(R192G) as mucosal adjuvants Neurobiol Aging 2002 23 991 1000 10.1016/s0197-4580(02)00127-6 12470794

[B68] McLean CA Cherny RA Fraser FW Fuller SJ Smith MJ Beyreuther K Bush AI Masters CL Soluble pool of Abeta amyloid as a determinant of severity of neurodegeneration in Alzheimer's disease Ann Neurol 1999 46 860 866 10.1002/1531-8249(199912)46:6<860::aid-ana8>3.0.co;2-m 10589538

[B69] Lue LF Kuo YM Roher AE Brachova L Shen Y Sue L Beach T Kurth JH Rydel RE Rogers J Soluble amyloid beta peptide concentration as a predictor of synaptic change in Alzheimer's disease Am J Pathol 1999 155 853 862 10.1016/s0002-9440(10)65184-x PMC1866907 10487842

[B70] Wang J Dickson DW Trojanowski JQ Lee VM The levels of soluble versus insoluble brain Abeta distinguish Alzheimer's disease from normal and pathologic aging Exp Neurol 1999 158 328 337 10.1006/exnr.1999.7085 10415140

[B71] Kuo YM Emmerling MR Vigo-Pelfrey C Kasunic TC Kirkpatrick JB Murdoch GH Ball MJ Roher AE Water-soluble Abeta (N-40, N-42) oligomers in normal and Alzheimer disease brains J Biol Chem 1996 271 4077 4081 10.1074/jbc.271.8.4077 8626743

[B72] Stenh C Englund H Lord A Johansson AS Almeida CG Gellerfors P Greengard P Gouras GK Lannfelt L Nilsson LN Amyloid-beta oligomers are inefficiently measured by enzyme-linked immunosorbent assay Ann Neurol 2005 58 147 150 10.1002/ana.20524 15984012

[B73] Morishima-Kawashima M Ihara Y The presence of amyloid β-protein in the detergent-insoluble membrane compartment of human neuroblastoma cells Biochem 1998 37 15247 15253 10.1021/bi981843u 9799484

[B74] Enya M Morishima-Kawashima M Yoshimura M Shinkai Y Kusui K Khan K Games D Schenk D Sugihara S Yamaguchi H Ihara Y Appearance of sodium dodecyl sulfate-stable amyloid beta-protein (Abeta) dimer in the cortex during aging Am J Pathol 1999 154 271 279 10.1016/s0002-9440(10)65273-x PMC1853431 9916941

[B75] Funato H Enya M Yoshimura M Morishima-Kawashima M Ihara Y Presence of sodium dodecyl sulfate-stable amyloid beta-protein dimers in the hippocampus CA1 not exhibiting neurofibrillary tangle formation Am J Pathol 1999 155 23 28 10.1016/s0002-9440(10)65094-8 PMC1866667 10393832

[B76] Englund H Degerman Gunnarsson M Brundin RM Hedlund M Kilander L Lannfelt L Ekholm Pettersson F Oligomerization Partially Explains the Lowering of Abeta42 in Alzheimer's Disease Cerebrospinal Fluid Neurodegener Dis 2009 6 139 147 10.1159/000225376 19521063

[B77] Shankar GM Li S Mehta TH Garcia-Munoz A Shepardson NE Smith I Brett FM Farrell MA Rowan MJ Lemere CA Regan CM Walsh DM Sabatini BL Selkoe DJ Amyloid-beta protein dimers isolated directly from Alzheimer's brains impair synaptic plasticity and memory Nat Med 2008 14 837 842 10.1038/nm1782 PMC2772133 18568035

[B78] Vigo-Pelfrey C Lee D Keim PS Lieberburg I Schenk D Characterization of beta-amyloid peptide from human cerebrospinal fluid Journal Neurochemistry 1993 61 1965 1968 10.1111/j.1471-4159.1993.tb09841.x 8229004

[B79] Roher A Wolfe D Palutke M KuKuruga D Purification, ultrastructure, and chemical analyses of Alzheimer's disease amyloid plaque core protein Proc Natl Acad Sci USA 1986 83 2662 2666 10.1073/pnas.83.8.2662 PMC323359 3458224

[B80] Ashe KH Learning and memory in transgenic mice modeling Alzheimer's disease Learn Mem 2001 8 301 308 10.1101/lm.43701 11773429

[B81] Games D Buttini M Kobayashi D Schenk D Seubert P Mice as models: transgenic approaches and Alzheimer's disease J Alzheimers Dis 2006 9 133 149 10.3233/jad-2006-9s316 16914852

[B82] Mucke L Masliah E Yu GQ Mallory M Rockenstein EM Tatsuno G Hu K Kholodenko D Johnson-Wood K McConlogue L High-level neuronal expression of abeta 1-42 in wild-type human amyloid protein precursor transgenic mice: synaptotoxicity without plaque formation J Neurosci 2000 20 4050 4058 10.1523/JNEUROSCI.20-11-04050.2000 PMC6772621 10818140

[B83] Hsia AY Masliah E McConlogue L Yu GQ Tatsuno G Hu K Kholodenko D Malenka RC Nicoll RA Mucke L Plaque-independent disruption of neural circuits in Alzheimer's disease mouse models Proc Natl Acad Sci USA 1999 96 3228 3233 10.1073/pnas.96.6.3228 PMC15924 10077666

[B84] Westerman MA Cooper-Blacketer D Mariash A Kotilinek L Kawarabayashi T Younkin LH Carlson GA Younkin SG Ashe KH The relationship between Abeta and memory in the Tg2576 mouse model of Alzheimer's disease J Neurosci 2002 22 1858 1867 10.1523/JNEUROSCI.22-05-01858.2002 PMC6758862 11880515

[B85] Kawarabayashi T Shoji M Younkin LH Wen-Lang L Dickson DW Murakami T Matsubara E Abe K Ashe KH Younkin SG Dimeric amyloid beta protein rapidly accumulates in lipid rafts followed by apolipoprotein E and phosphorylated tau accumulation in the Tg2576 mouse model of Alzheimer's disease J Neurosci 2004 24 3801 3809 10.1523/JNEUROSCI.5543-03.2004 PMC6729359 15084661

[B86] Lesne S Koh MT Kotilinek L Kayed R Glabe CG Yang A Gallagher M Ashe KH A specific amyloid-beta protein assembly in the brain impairs memory Nature 2006 440 352 357 10.1038/nature04533 16541076

[B87] Cheng IH Scearce-Levie K Legleiter J Palop JJ Gerstein H Bien-Ly N Puolivali J Lesne S Ashe KH Muchowski PJ Mucke L Accelerating amyloid-beta fibrillization reduces oligomer levels and functional deficits in Alzheimer disease mouse models J Biol Chem 2007 282 23818 23828 10.1074/jbc.M701078200 17548355

[B88] Dodart JC Bales KR Gannon KS Greene SJ DeMattos RB Mathis C DeLong CA Wu S Wu X Holtzman DM Paul SM Immunization reverses memory deficits without reducing brain Abeta burden in Alzheimer's disease model Nat Neurosci 2002 5 452 457 10.1038/nn842 11941374

[B89] Reed MN Hofmeister JJ Jungbauer L Welzel AT Yu C Lesné S LaDu MJ Walsh DM Ashe KH Cleary JP Cognitive Effects of Naturally and Synthetically Derived A^® ^Oligomers Neurobiology of Aging 2009 in press 10.1016/j.neurobiolaging.2009.11.007 PMC2895944 20031278

[B90] Dineley KT Xia XF Bui D Sweatt JD Zheng H Accelerated plaque accumulation, associative learning deficits, and up-regulation of alpha 7 nicotinic receptor protein in transgenic mice co-expressing mutant human presenilin 1 and amyloid precursor proteins Journal of Biological Chemistry 2002 277 22768 22780 10.1074/jbc.M200164200 11912199

[B91] Jacobsen JS Wu CC Redwine JM Comery TA Arias R Bowlby M Martone R Morrison JH Pangalos MN Reinhart PH Bloom FE Early-onset behavioral and synaptic deficits in a mouse model of Alzheimer's disease Proc Natl Acad Sci USA 2006 103 5161 5166 10.1073/pnas.0600948103 PMC1405622 16549764

[B92] Shankar GM Leissring MA Adame A Sun X Spooner E Masliah E Selkoe DJ Lemere CA Walsh DM Biochemical and immunohistochemical analysis of an Alzheimer's disease mouse model reveals the presence of multiple cerebral Abeta assembly forms throughout life Neurobiol Dis 2009 36 293 302 10.1016/j.nbd.2009.07.021 PMC2782414 19660551

[B93] Meilandt WJ Cisse M Ho K Wu T Esposito LA Scearce-Levie K Cheng IH Yu GQ Mucke L Neprilysin overexpression inhibits plaque formation but fails to reduce pathogenic Abeta oligomers and associated cognitive deficits in human amyloid precursor protein transgenic mice J Neurosci 2009 29 1977 1986 10.1523/JNEUROSCI.2984-08.2009 PMC2768427 19228952

[B94] Li S Hong S Shepardson NE Walsh DM Shankar GM Selkoe D Soluble oligomers of amyloid Beta protein facilitate hippocampal long-term depression by disrupting neuronal glutamate uptake Neuron 2009 62 788 801 10.1016/j.neuron.2009.05.012 PMC2702854 19555648

[B95] Podlisny MB Ostaszewski BL Squazzo SL Koo EH Rydell RE Teplow DB Selkoe DJ Aggregation of secreted amyloid beta-protein into sodium dodecyl sulfate-stable oligomers in cell culture J Biol Chem 1995 270 9564 9570 10.1074/jbc.270.16.9564 7721886

[B96] Xia W Zhang J Kholodenko D Citron M Podlisny MB Teplow DB Haass C Seubert P Koo EH Selkoe DJ Enhanced production and oligomerization of the 42-residue amyloid beta-protein by Chinese hamster ovary cells stably expressing mutant presenilins Journal Biological Chemistry 1997 272 7977 7982 10.1074/jbc.272.12.7977 9065468

[B97] Walsh DM Tseng BP Rydel RE Podlisny MB Selkoe DJ The oligomerization of amyloid beta-protein begins intracellularly in cells derived from human brain Biochemistry 2000 39 10831 10839 10.1021/bi001048s 10978169

[B98] Townsend M Shankar GM Mehta T Walsh DM Selkoe DJ Effects of secreted oligomers of amyloid {beta}-protein on hippocampal synaptic plasticity: a potent role for trimers J Physiol 2006 572 477 492 10.1113/jphysiol.2005.103754 PMC1779683 16469784

[B99] Poling A Morgan-Paisley K Panos JJ Kim EM O'Hare E Cleary JP Lesne S Ashe KH Porritt M Baker LE Oligomers of the amyloid-beta protein disrupt working memory: confirmation with two behavioral procedures Behav Brain Res 2008 193 230 234 10.1016/j.bbr.2008.06.001 PMC2786170 18585407

[B100] Calabrese B Shaked GM Tabarean IV Braga J Koo EH Halpain S Rapid, concurrent alterations in pre-and postsynaptic structure induced by naturally-secreted amyloid-beta protein Mol Cell Neurosci 2007 35 183 193 10.1016/j.mcn.2007.02.006 PMC2268524 17368908

[B101] Walsh DM Klyubin I Fadeeva JV Rowan MJ Selkoe DJ Amyloid-beta oligomers: their production, toxicity and therapeutic inhibition Biochem Soc Trans 2002 30 552 557 10.1042/bst0300552 12196135

[B102] Walsh D Klyubin I Fadeeva J William K Cullen W Anwyl R Wolfe M Rowan M Selkoe D Naturally secreted oligomers of the Alzheimer amyloid beta-protein potently inhibit hippocampal long-term potentiation *in vivo* Nature 2002 416 535 539 10.1038/416535a 11932745

[B103] Klyubin I Betts V Welzel AT Blennow K Zetterberg H Wallin A Lemere CA Cullen WK Peng Y Wisniewski T Selkoe DJ Anwyl R Walsh DM Rowan MJ Amyloid beta protein dimer-containing human CSF disrupts synaptic plasticity: prevention by systemic passive immunization J Neurosci 2008 28 4231 4237 10.1523/JNEUROSCI.5161-07.2008 PMC2685151 18417702

[B104] Wang Q Walsh DM Rowan MJ Selkoe DJ Anwyl R Block of long-term potentiation by naturally secreted and synthetic amyloid beta-peptide in hippocampal slices is mediated via activation of the kinases c-Jun N-terminal kinase, cyclin-dependent kinase 5, and p38 mitogen-activated protein kinase as well as metabotropic glutamate receptor type 5 J Neurosci 2004 24 3370 3378 10.1523/JNEUROSCI.1633-03.2004 PMC6730034 15056716

[B105] Walsh DM Townsend M Podlisny MB Shankar GM Fadeeva JV Agnaf OE Hartley DM Selkoe DJ Certain inhibitors of synthetic amyloid beta-peptide (Abeta) fibrillogenesis block oligomerization of natural Abeta and thereby rescue long-term potentiation J Neurosci 2005 25 2455 2462 10.1523/JNEUROSCI.4391-04.2005 PMC6725159 15758153

[B106] Shankar GM Bloodgood BL Townsend M Walsh DM Selkoe DJ Sabatini BL Natural oligomers of the Alzheimer amyloid-beta protein induce reversible synapse loss by modulating an NMDA-type glutamate receptor-dependent signaling pathway J Neurosci 2007 27 2866 2875 10.1523/JNEUROSCI.4970-06.2007 PMC6672572 17360908

[B107] Cleary JP Walsh DM Hofmeister JJ Shankar GM Kuskowski MA Selkoe DJ Ashe KH Natural oligomers, but not monomers, of the amyloid β-protein specifically disrupt the memory of learned behavior Nature Neuroscience 2005 8 79 84 10.1038/nn1372 15608634

[B108] Hartley DM Walsh DM Ye CP Diehl T Vasquez S Vassilev PM Teplow DB Selkoe DJ Protofibrillar intermediates of amyloid β-protein induce acute electrophysiological changes and progressive neurotoxicity in cortical neurons J Neurosci 1999 19 8876 8884 10.1523/JNEUROSCI.19-20-08876.1999 PMC6782787 10516307

[B109] Wang HW Pasternak JF Kuo H Ristic H Lambert MP Chromy B Viola KL Klein WL Stine WB Krafft GA Trommer BL Soluble oligomers of beta-amyloid(1-42) inhibit long-term potentiation but not long-term depression in rat dentate gyrus Brain Research 2002 924 133 140 10.1016/s0006-8993(01)03058-x 11750898

[B110] Shrestha BR Vitolo OV Joshi P Lordkipanidze T Shelanski M Dunaevsky A Amyloid beta peptide adversely affects spine number and motility in hippocampal neurons Mol Cell Neurosci 2006 33 274 282 10.1016/j.mcn.2006.07.011 16962789

[B111] Lacor PN Buniel MC Furlow PW Clemente AS Velasco PT Wood M Viola KL Klein WL Abeta oligomer-induced aberrations in synapse composition, shape, and density provide a molecular basis for loss of connectivity in Alzheimer's disease J Neurosci 2007 27 796 807 10.1523/JNEUROSCI.3501-06.2007 PMC6672917 17251419

[B112] Walsh DM Thulin E Minogue AM Gustavsson N Pang E Teplow DB Linse S A facile method for expression and purification of the Alzheimer's disease-associated amyloid beta-peptide FEBS J 2009 276 1266 1281 10.1111/j.1742-4658.2008.06862.x PMC2702495 19175671

[B113] Harper JD Wong SS Lieber CM Lansbury PT Assembly of Aβ amyloid protofibrils: An *in vitro *model for a possible early event in Alzheimer's disease Biochemistry 1999 38 8972 8980 10.1021/bi9904149 10413470

[B114] Lashuel HA Hartley D Petre BM Walz T Lansbury PT Jr Neurodegenerative disease: amyloid pores from pathogenic mutations Nature 2002 418 291 10.1038/418291a 12124613

[B115] Isaacs AM Senn DB Yuan M Shine JP Yankner BA Acceleration of amyloid beta-peptide aggregation by physiological concentrations of calcium J Biol Chem 2006 281 27916 27923 10.1074/jbc.M602061200 PMC1595535 16870617

[B116] Hartley DM Zhao C Speier AC Woodard GA Li S Li Z Walz T Transglutaminase induces protofibril-like amyloid beta-protein assemblies that are protease-resistant and inhibit long-term potentiation J Biol Chem 2008 283 16790 16800 10.1074/jbc.M802215200 PMC2423271 18397883

[B117] Martins IC Kuperstein I Wilkinson H Maes E Vanbrabant M Jonckheere W Van Gelder P Hartmann D D'Hooge R De Strooper B Schymkowitz J Rousseau F Lipids revert inert Abeta amyloid fibrils to neurotoxic protofibrils that affect learning in mice EMBO J 2008 27 224 233 10.1038/sj.emboj.7601953 PMC2206134 18059472

[B118] Walsh DM Lomakin A Benedek GB Maggio JE Condron MM Teplow DB Amyloid β-protein fibrillogenesis: Detection of a protofibrillar intermediate J Biol Chem 1997 272 22364 22374 10.1074/jbc.272.35.22364 9268388

[B119] Nybo M Svehag SE Holm Nielsen E An ultrastructural study of amyloid intermediates in A beta1-42 fibrillogenesis Scand J Immunol 1999 49 219 223 10.1046/j.1365-3083.1999.00526.x 10102637

[B120] Hepler RW Grimm KM Nahas DD Breese R Dodson EC Acton P Keller PM Yeager M Wang H Shughrue P Kinney G Joyce JG Solution state characterization of amyloid beta-derived diffusible ligands Biochemistry 2006 45 15157 15167 10.1021/bi061850f 17176037

[B121] Lauren J Gimbel DA Nygaard HB Gilbert JW Strittmatter SM Cellular prion protein mediates impairment of synaptic plasticity by amyloid-beta oligomers Nature 2009 457 1128 1132 10.1038/nature07761 PMC2748841 19242475

[B122] Dahlgren KN Manelli AM Stine WB Jr Baker LK Krafft GA LaDu MJ Oligomeric and fibrillar species of amyloid-β peptides differentially affect neuronal viability J Biol Chem 2002 277 32046 32053 10.1074/jbc.M201750200 12058030

[B123] Kim HJ Chae SC Lee DK Chromy B Lee SC Park YC Klein WL Krafft GA Hong ST Selective neuronal degeneration induced by soluble oligomeric amyloid beta protein Faseb J 2003 17 118 120 10.1096/fj.01-0987fje 12424218

[B124] Lacor PN Buniel MC Chang L Fernandez SJ Gong Y Viola KL Lambert MP Velasco PT Bigio EH Finch CE Krafft GA Klein WL Synaptic targeting by Alzheimer's-related amyloid beta oligomers J Neurosci 2004 24 10191 10200 10.1523/JNEUROSCI.3432-04.2004 PMC6730194 15537891

[B125] Gong Y Chang L Viola KL Lacor PN Lambert MP Finch CE Krafft GA Klein WL Alzheimer's disease-affected brain: presence of oligomeric Abeta ligands (ADDLs) suggests a molecular basis for reversible memory loss Proceedings of the National Academy of Sciences (USA) 2003 100 10417 10422 10.1073/pnas.1834302100 PMC193576 12925731

[B126] Lambert MP Viola KL Chromy BA Chang L Morgan TE Yu J Venton DL Krafft GA Finch CE Klein WL Vaccination with soluble Abeta oligomers generates toxicity-neutralizing antibodies J Neurochem 2001 79 595 605 10.1046/j.1471-4159.2001.00592.x 11701763

[B127] Chang L Bakhos L Wang ZG Venton DL Klein WL Femtomole Immunodetection of synthetic and endogenous amyloid-b oligomers and its application to Alzheimer's disease drug candidate screening Journal of Molecular Neuroscience 2003 20 305 313 10.1385/JMN:20:3:305 14501013

[B128] Kayed R Head E Thompson JL McIntire TM Milton SC Cotman CW Glabe CG Common structure of soluble amyloid oligomers implies common mechanism of pathogenesis Science 2003 300 486 489 10.1126/science.1079469 12702875

[B129] Whalen BM Selkoe DJ Hartley DM Small non-fibrillar assemblies of amyloid beta-protein bearing the Arctic mutation induce rapid neuritic degeneration Neurobiol Dis 2005 20 254 266 10.1016/j.nbd.2005.03.007 16242634

[B130] Maloney MT Minamide LS Kinley AW Boyle JA Bamburg JR Beta-secretase-cleaved amyloid precursor protein accumulates at actin inclusions induced in neurons by stress or amyloid beta: a feedforward mechanism for Alzheimer's disease J Neurosci 2005 25 11313 11321 10.1523/JNEUROSCI.3711-05.2005 PMC6725891 16339026

[B131] Kelly BL Ferreira A beta-Amyloid-induced dynamin 1 degradation is mediated by N-methyl-D-aspartate receptors in hippocampal neurons J Biol Chem 2006 281 28079 28089 10.1074/jbc.M605081200 16864575

[B132] Barghorn S Zheng-Fischhofer Q Ackmann M Biernat J von Bergen M Mandelkow E Structure, microtubule interactions, and paired helical filament aggregation by tau mutants of frontotemporal dementias [In Process Citation] Biochemistry 2000 39 11714 11721 10.1021/bi000850r 10995239

[B133] Deshpande A Mina E Glabe C Busciglio J Different conformations of amyloid beta induce neurotoxicity by distinct mechanisms in human cortical neurons J Neurosci 2006 26 6011 6018 10.1523/JNEUROSCI.1189-06.2006 PMC6675207 16738244

[B134] Demuro A Mina E Kayed R Milton SC Parker I Glabe CG Calcium dysregulation and membrane disruption as a ubiquitous neurotoxic mechanism of soluble amyloid oligomers J Biol Chem 2005 280 17294 17300 10.1074/jbc.M500997200 15722360

[B135] Wogulis M Wright S Cunningham D Chilcote T Powell K Rydel RE Nucleation-dependent polymerization is an essential component of amyloid-mediated neuronal cell death J Neurosci 2005 25 1071 1080 10.1523/JNEUROSCI.2381-04.2005 PMC6725948 15689542

[B136] White AR Reyes R Mercer JF Camakaris J Zheng H Bush AI Multhaup G Beyreuther K Masters CL Cappai R Copper levels are increased in the cerebral cortex and liver of APP and APLP2 knockout mice Brain Research 1999 842 439 444 10.1016/s0006-8993(99)01861-2 10526140

[B137] Snyder EM Nong Y Almeida CG Paul S Moran T Choi EY Nairn AC Salter MW Lombroso PJ Gouras GK Greengard P Regulation of NMDA receptor trafficking by amyloid-beta Nat Neurosci 2005 8 1051 1058 10.1038/nn1503 16025111

[B138] Tamagno E Bardini P Guglielmotto M Danni O Tabaton M The various aggregation states of beta-amyloid 1-42 mediate different effects on oxidative stress, neurodegeneration, and BACE-1 expression Free Radic Biol Med 2006 41 202 212 10.1016/j.freeradbiomed.2006.01.021 16814100

[B139] Zhao L Ma QL Calon F Harris-White ME Yang F Lim GP Morihara T Ubeda OJ Ambegaokar S Hansen JE Weisbart RH Teter B Frautschy SA Cole GM Role of p21-activated kinase pathway defects in the cognitive deficits of Alzheimer disease Nat Neurosci 2006 9 234 242 10.1038/nn1630 16415866

[B140] Tomiyama T Nagata T Shimada H Teraoka R Fukushima A Kanemitsu H Takuma H Kuwano R Imagawa M Ataka S Wada Y Yoshioka E Nishizaki T Watanabe Y Mori H A new amyloid beta variant favoring oligomerization in Alzheimer's-type dementia Ann Neurol 2008 63 377 387 10.1002/ana.21321 18300294

[B141] Hung LW Ciccotosto GD Giannakis E Tew DJ Perez K Masters CL Cappai R Wade JD Barnham KJ Amyloid-beta peptide (Abeta) neurotoxicity is modulated by the rate of peptide aggregation: Abeta dimers and trimers correlate with neurotoxicity J Neurosci 2008 28 11950 11958 10.1523/JNEUROSCI.3916-08.2008 PMC6671645 19005060

[B142] Harmeier A Wozny C Rost BR Munter LM Hua H Georgiev O Beyermann M Hildebrand PW Weise C Schaffner W Schmitz D Multhaup G Role of amyloid-beta glycine 33 in oligomerization, toxicity, and neuronal plasticity J Neurosci 2009 29 7582 7590 10.1523/JNEUROSCI.1336-09.2009 PMC6665404 19515926

[B143] Kamenetz F Tomita T Hsieh H Seabrook G Borchelt D Iwatsubo T Sisodia S Malinow R APP processing and synaptic function Neuron 2003 37 925 937 10.1016/s0896-6273(03)00124-7 12670422

[B144] Brody DL Magnoni S Schwetye KE Spinner ML Esparza TJ Stocchetti N Zipfel GJ Holtzman DM Amyloid-beta dynamics correlate with neurological status in the injured human brain Science 2008 321 1221 1224 10.1126/science.1161591 PMC2577829 18755980

[B145] Hsieh H Boehm J Sato C Iwatsubo T Tomita T Sisodia S Malinow R AMPAR removal underlies Abeta-induced synaptic depression and dendritic spine loss Neuron 2006 52 831 843 10.1016/j.neuron.2006.10.035 PMC1850952 17145504

[B146] Plant LD Boyle JP Smith IF Peers C Pearson HA The production of amyloid beta peptide is a critical requirement for the viability of central neurons J Neurosci 2003 23 5531 5535 10.1523/JNEUROSCI.23-13-05531.2003 PMC6741264 12843253

[B147] Puzzo D Privitera L Leznik E Fa M Staniszewski A Palmeri A Arancio O Picomolar amyloid-beta positively modulates synaptic plasticity and memory in hippocampus J Neurosci 2008 28 14537 14545 10.1523/JNEUROSCI.2692-08.2008 PMC2673049 19118188

[B148] Palop JJ Chin J Roberson ED Wang J Thwin MT Bien-Ly N Yoo J Ho KO Yu GQ Kreitzer A Finkbeiner S Noebels JL Mucke L Aberrant excitatory neuronal activity and compensatory remodeling of inhibitory hippocampal circuits in mouse models of Alzheimer's disease Neuron 2007 55 697 711 10.1016/j.neuron.2007.07.025 PMC8055171 17785178

[B149] Busche MA Eichhoff G Adelsberger H Abramowski D Wiederhold KH Haass C Staufenbiel M Konnerth A Garaschuk O Clusters of hyperactive neurons near amyloid plaques in a mouse model of Alzheimer's disease Science 2008 321 1686 1689 10.1126/science.1162844 18802001

[B150] Spires TL Meyer-Luehmann M Stern EA McLean PJ Skoch J Nguyen PT Bacskai BJ Hyman BT Dendritic spine abnormalities in amyloid precursor protein transgenic mice demonstrated by gene transfer and intravital multiphoton microscopy J Neurosci 2005 25 7278 7287 10.1523/JNEUROSCI.1879-05.2005 PMC1820616 16079410

[B151] Wang HY Lee DH Davis CB Shank RP Amyloid peptide Abeta(1-42) binds selectively and with picomolar affinity to alpha7 nicotinic acetylcholine receptors J Neurochem 2000 75 1155 1161 10.1046/j.1471-4159.2000.0751155.x 10936198

[B152] Dewachter I Filipkowski RK Priller C Ris L Neyton J Croes S Terwel D Gysemans M Devijver H Borghgraef P Godaux E Kaczmarek L Herms J Van Leuven F Deregulation of NMDA-receptor function and down-stream signaling in APP[V717I] transgenic mice Neurobiol Aging 2009 30 241 256 10.1016/j.neurobiolaging.2007.06.011 17673336

[B153] Yang SN Tang YG Zucker RS Selective induction of LTP and LTD by postsynaptic [Ca2+]i elevation J Neurophysiol 1999 81 781 787 10.1152/jn.1999.81.2.781 10036277

[B154] Neveu D Zucker RS Postsynaptic levels of [Ca2+]i needed to trigger LTD and LTP Neuron 1996 16 619 629 10.1016/s0896-6273(00)80081-1 8785059

[B155] Ye C Walsh DM Selkoe DJ Hartley DM Amyloid beta-protein induced electrophysiological changes are dependent on aggregation state: N-methyl-D-aspartate (NMDA) versus non-NMDA receptor/channel activation Neurosci Lett 2004 366 320 325 10.1016/j.neulet.2004.05.060 15288443

[B156] Snyder EM Philpot BD Huber KM Dong X Fallon JR Bear MF Internalization of ionotropic glutamate receptors in response to mGluR activation Nat Neurosci 2001 4 1079 1085 10.1038/nn746 11687813

[B157] Zakharenko SS Zablow L Siegelbaum SA Altered presynaptic vesicle release and cycling during mGluR-dependent LTD Neuron 2002 35 1099 1110 10.1016/s0896-6273(02)00898-x 12354399

[B158] O'Shea SD Smith IM McCabe OM Walsh DM O'Connor WT Intracerebroventricular administration of amyloid β-protein oligomers selectively increases dorsal hippocampal dialysate glutamate levels in the awake rat Sensors 2008 8 7428 7437 10.3390/s8117428 PMC3787453 27873937

[B159] Zhao WQ De Felice FG Fernandez S Chen H Lambert MP Quon MJ Krafft GA Klein WL Amyloid beta oligomers induce impairment of neuronal insulin receptors FASEB J 2008 22 246 260 10.1096/fj.06-7703com 17720802

[B160] Lashuel HA Hartley DM Petre BM Wall JS Simon MN Walz T Lansbury PT Jr Mixtures of wild-type and a pathogenic (E22G) form of Abeta40 in vitro accumulate protofibrils, including amyloid pores J Mol Biol 2003 332 795 808 10.1016/s0022-2836(03)00927-6 12972252

[B161] Yu L Edalji R Harlan JE Holzman TF Lopez AP Labkovsky B Hillen H Barghorn S Ebert U Richardson PL Miesbauer L Solomon L Bartley D Walter K Johnson RW Hajduk PJ Olejniczak ET Structural characterization of a soluble amyloid beta-peptide oligomer Biochemistry 2009 48 1870 1877 10.1021/bi802046n 19216516

[B162] Barghorn S Nimmrich V Striebinger A Krantz C Keller P Janson B Bahr M Schmidt M Bitner RS Harlan J Barlow E Ebert U Hillen H Globular amyloid beta-peptide oligomer-a homogenous and stable neuropathological protein in Alzheimer's disease J Neurochem 2005 95 834 847 10.1111/j.1471-4159.2005.03407.x 16135089

[B163] Hermann D Both M Ebert U Gross G Schoemaker H Draguhn A Wicke K Nimmrich V Synaptic transmission is impaired prior to plaque formation in amyloid precursor protein-overexpressing mice without altering behaviorally-correlated sharp wave-ripple complexes Neuroscience 2009 162 1081 1090 10.1016/j.neuroscience.2009.05.044 19477243

[B164] Noguchi A Matsumura S Dezawa M Tada M Yanazawa M Ito A Akioka M Kikuchi S Sato M Ideno S Noda M Fukunari A Muramatsu SI Itokazu Y Sato K Takahashi H Teplow DB Nabeshima YI Kakita A Imahori K Hoshi M Isolation and characterization of patient-derived, toxic, high-mass amyloid {beta}-protein (A{beta}) assembly from Alzheimer's disease brains J Biol Chem 2009 47 32895 905 10.1074/jbc.M109.000208 PMC2781705 19759000

[B165] Hu NW Smith IM Walsh DM Rowan MJ Soluble amyloid-beta peptides potently disrupt hippocampal synaptic plasticity in the absence of cerebrovascular dysfunction in vivo Brain 2008 131 2414 2424 10.1093/brain/awn174 18678563

